# Ultra-doped *n*-type germanium thin films for sensing in the mid-infrared

**DOI:** 10.1038/srep27643

**Published:** 2016-06-10

**Authors:** Slawomir Prucnal, Fang Liu, Matthias Voelskow, Lasse Vines, Lars Rebohle, Denny Lang, Yonder Berencén, Stefan Andric, Roman Boettger, Manfred Helm, Shengqiang Zhou, Wolfgang Skorupa

**Affiliations:** 1 Helmholtz-Zentrum Dresden-Rossendorf, Institute of Ion Beam Physics and Materials Research, Bautzner Landstrasse 400, 01328 Dresden, Germany; 2 Department of Physics/Centre for Materials Science and Nanotechnology, University of Oslo, P.O. Box 1048 Blindern, N-0316 Oslo, Norway; 3 Center for Advancing Electronics Dresden (cfaed), Technische Universität Dresden, 01062 Dresden, Germany

## Abstract

A key milestone for the next generation of high-performance multifunctional microelectronic devices is the monolithic integration of high-mobility materials with Si technology. The use of Ge instead of Si as a basic material in nanoelectronics would need homogeneous *p*- and *n*-type doping with high carrier densities. Here we use ion implantation followed by rear side flash-lamp annealing (r-FLA) for the fabrication of heavily doped *n*-type Ge with high mobility. This approach, in contrast to conventional annealing procedures, leads to the full recrystallization of Ge films and high P activation. In this way single crystalline Ge thin films free of defects with maximum attained carrier concentrations of 2.20 ± 0.11 × 10^20^ cm^−3^ and carrier mobilities above 260 cm^2^/(V·s) were obtained. The obtained ultra-doped Ge films display a room-temperature plasma frequency above 1,850 cm^−1^, which enables to exploit the plasmonic properties of Ge for sensing in the mid-infrared spectral range.

The incorporation of different functional optoelectronic elements on a single chip enables performance progress, which can overcome the downsizing limit in Si technology. To this day the application of Ge is limited to fiber-optic systems, infrared optics, photovoltaics and special applications in microelectronics where the high performance logic devices are crucial^1^,^2^,^3^,^4^,^5^,^6^,^7^,^8^. Furthermore, plasmonic sensing in the mid-infrared (MIR) has not been accessible to semiconductors so far, however, ultra-doped *n*-type Ge with carrier concentration in the range of 10^19^ to 10^21 ^cm^−3^ is perfectly suited for such purpose^9^,^10^,^11^. When compared with *n*-type Si, *n*-type Ge is characterised by a smaller electron effective mass, 0.12 m_e_ for Ge vs. 0.26 m_e_ for Si, which corresponds to a higher plasma frequency (ω_p_) for certain doping levels. The near-infrared and MIR is the optical spectral range where most of the vibrational modes of bio- and gas molecules are located. The use of group IV-semiconductors for the plasmonic sensors will increase the diversity of microelectronic device applications fabricated in the Si complementary metal-oxide semiconductor (CMOS) technology processes^12^,^13^. For example, nanostructured *n*-type Ge with an ultra-high doping level can replace nanostructured gold antennas used nowadays^10^. Moreover, the fabrication of ultra-doped *n*-type Ge in a controllable way allows for the conversion of Ge from an indirect to a direct bandgap semiconductor via decreasing the energy difference between the conduction band valleys at the Γ and L point and can finally introduce a crossover of the Γ and L conduction band minima^14^,^15^. One of the main obstacles towards application of Ge in plasmonics is the lack of an efficient doping method for the fabrication of heavily doped Ge n-type layers with effective carrier concentrations well above 10^19 ^cm^−3^.

In this paper we report on a sub-second fast annealing method using flash lamp annealing (FLA) to recrystallize Ge layers amorphized during ion implantation and to activate P dopants. It is shown that P implanted Ge with an amorphous layer thickness above 200 nm can be completely recrystallized without a significant diffusion of P. The novelty of our approach is based on the millisecond range *rear-side* FLA (r-FLA) treatment. Using r-FLA an effective carrier concentration above 2 × 10^20 ^cm^−3^ with an activation efficiency around 80% and a carrier mobility above 260 cm^2^/(V·s) in P doped Ge is achieved. Such high activation efficiency in ultra-doped Ge makes ion implantation followed by r-FLA unique among different doping techniques. The recrystallization mechanism of Ge during millisecond range r-FLA, namely the interplay between random nucleation and solid phase epitaxy (SPE), is discussed in detail. We also demonstrate a room-temperature plasma frequency as high as 1,850 cm^−1^, which is suited for mid-infrared plasmonics.

## Results

### Structural properties

In fact, *n*-type doping of Ge is a key bottleneck in the realization of advanced negative-channel metal-oxide-semiconductor (NMOS) devices^16^. The donors are fast diffusers and the diffusion is known to be mediated by vacancies^17^. Moreover, the energy levels of vacancy defects in Ge are located near the valence band edge leading to significant compensation of the *n*-type dopants like P or As^18^. Finally, the maximum effective carrier concentration in P doped Ge is limited by the self-compensation effect and by the P out-diffusion during growth which again limits the electrical activation efficiency of P to about 20% for doping above the solid-solubility limit (above 2 × 10^20 ^cm^−3^)^19^,^20^,^21^. Therefore, single-step ion implantation followed by conventional annealing only allows for *n*-type doping in Ge in the range of 1–3 × 10^19 ^cm^−3^. Using millisecond-range flash lamp annealing (FLA) and ion implantation *n*-type and *p*-type Ge layers with an active carrier concentration above 6 × 10^19 ^cm^−3^ and 3 × 10^20 ^cm^−3^ were reported, respectively^22^,^23^,^24^. Also, a similar electron concentration was obtained by the *in-situ* doping of epitaxial Ge thin films during chemical vapour deposition (CVD) or standard molecular beam epitaxy (MBE) using *in-diffusion* with a δ-doped layer as the source of P^25^,^26^.

**Figure 1:**
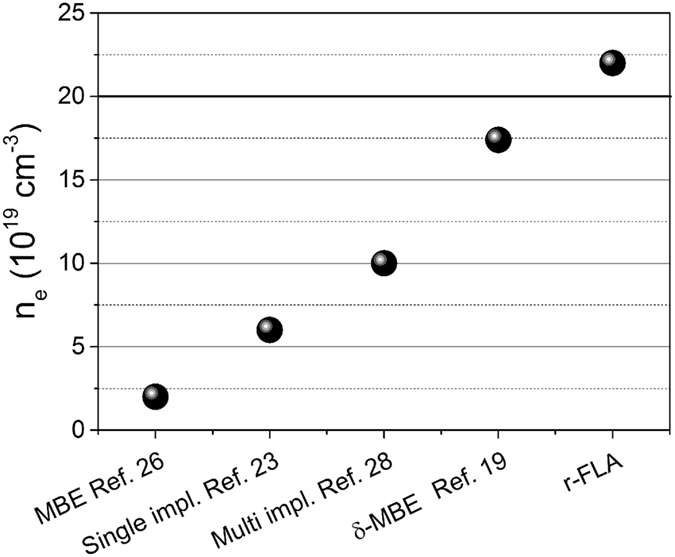
The maximum concentration of electrically active charge carriers in P doped Ge obtained by different techniques. Ion implantation followed by r-FLA refers to our Ge films, which show the highest active carrier concentration values ever reported so far. (See references for further details).

Recently, G. Scappucci, *et al*. have proposed a MBE based bottom-up approach to produce *n*-type metallic Ge layers with electrically active carrier concentrations as high as 1.9 × 10^20 ^cm^−3^ and 0.9 × 10^20 ^cm^−3^ in the bi-layer and multi-layer sample, respectively^19^,^20^. The recent progress in the *n*-type doping in Ge is summarized in Fig. 1.

In general, the doping of semiconductors can be performed either *in-situ*^26^, during the growth of thin films, or *ex-situ* where the ion implantation is the only non-equilibrium technique allowing to introduce impurities into semiconductors significantly above the solid solubility limit. To this day, the highest reported value for the density of electrically active donors in P doped Ge was 1.74 × 10^20 ^cm^−3^ with 44% of the electrically active dopants^19^,^27^. Such high dopant concentration was achieved by stacking multiple Ge:P δ-doped layers with 2 nm of Ge spacer-layer using MBE. In the case of P implanted Ge a doping level of 6 × 10^19 ^cm^−3^ was obtained using ultra-short FLA treatment in the millisecond range^23^ and 1 × 10^20 ^cm^−3^ by multi-implantation approach followed by rapid thermal annealing after each implantation step^28^.

**Figure 2:**
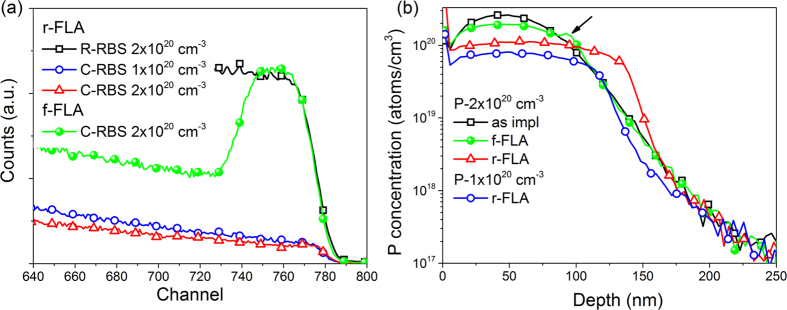
RBS random (R-RBS) and channelling (C-RBS) spectra obtained from samples implanted with P ions and annealed for 3 ms (**a**) and P distribution in as-implanted and annealed samples obtained by SIMS for two different P concentrations (**b**). Samples were annealed by both methods (f-FLA and r-FLA). The arrow in (**b**) indicates the polycrystalline/single crystalline transition and the peak at the surface is due to an artifact during SIMS measurements.

Figure 2a shows the Rutherford backscattering/channelling (RBS/C) spectra obtained from a P implanted Ge wafer after front FLA (f-FLA) and r-FLA. As already mentioned, ion implantation followed by millisecond range FLA has successfully been demonstrated to achieve a maximum carrier concentration in the range of 6 × 10^19 ^cm^−3^ for a P concentration above 10^21 ^cm^−3^ in Ge^23^. However, according to ref. 23 the implanted layer is epitaxially regrown after f-FLA for 3 ms, only if the damaged zone is thinner than 80 nm^23^. If the damaged zone is thicker than 80 nm, polycrystalline Ge is formed after the f-FLA. Such a behaviour has also been observed in our study (see green curve in Fig. 2a). Within a 115 nm thick surface layer (channel number 750 to 780), the random and channeling spectra obtained from the front-side flashed sample are almost identical. It implies that this region is either strongly disordered or polycrystalline. The thickness of the polycrystalline layer was extracted from the RBS raw data, using established RBS analysis software RUMP^29^. Additionally, from the P profile obtained by SIMS (see an arrow at green curve, Fig. 2b) a small peak located at around 110 nm from the surface is clearly observed in the f-FLA samples. This peak precisely coincides with the interface between the polycrystalline and the epitaxially regrown layer. Moreover, the channelling spectra recorded from rear-side flashed samples with two different P concentrations show no significant differences of the backscattering yield between the implanted depth and the single crystalline bulk Ge. This proves directly the formation of a single crystalline *n*-type Ge layer via solid phase epitaxy. Due to the slight diffusion of P atoms during r-FLA, the peak of the atomic concentration of P is reduced from 2 × 10^20 ^cm^−3^ (as-implanted) to 1.12 × 10^20 ^cm^−3^ after annealing as can be seen in Fig. 2b (red curve).

### Electrical characterisation of P doped Ge

**Figure 3:**
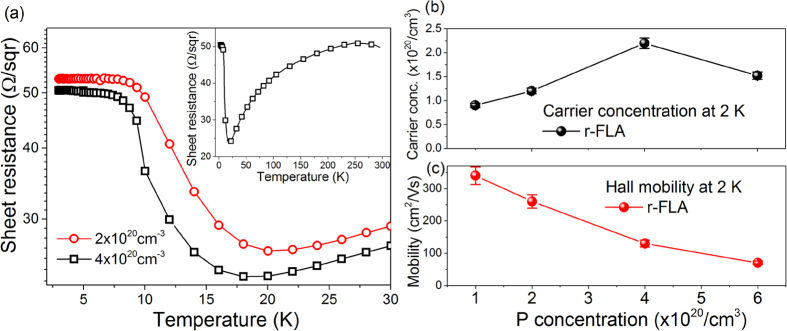
The sheet resistances of the Ge samples doped with the P concentrations of 2 × 10^20 ^cm^−3^ and 4 × 10^20 ^cm^−3^ after FLA in the low-temperature regime (**a**). The effective carrier concentration and the carrier mobility as a function of the P concentration are shown in (**b**,**c**), respectively. The inset in (**a**) shows the changes of sheet resistance over the temperature range of 2 to 300 K measured for the sample with a P peak concentration of 4 × 10^20 ^cm^−3^. All samples were annealed from the rear side with a flash energy of 61.5 Jcm^−1^.

The sheet resistance and Hall Effect measurements were performed in a 4-probe van der Pauw configuration in the temperature range from 2 to 300 K. Figure 3a shows the low-temperature range (below 30 K) of sheet resistance measured for two Ge samples after r-FLA with a nominal P concentration of 2 × 10^20 ^cm^−3^ and 4 × 10^20 ^cm^−3^ (the P concentration is referred to the as-implanted stage). The sheet resistance in the temperature range of 2 to 300 K is shown in the inset of Fig. 3a. Starting from 300 K it slightly decreases with decreasing temperature, goes through a minimum around 15 to 25 K, slowly increases and becomes temperature independent below 8 K. Taking into account the doped layer thickness of about 100 nm the mean layer resistivity of doped layer at low temperature is in the range of 10^−4^ Ωcm. Such a low resistivity and temperature independent sheet resistance below 8 K indicates that the P-doped Ge layers are metallic^20^. In order to eliminate an influence of the *p*-type substrate on the electrical properties of P doped Ge layers the Hall effect measurements presented in Fig. 3b,c were performed at 2 K. At such low temperatures, the carrier contribution from the substrate to the transport measurement can be neglected since acceptors are frozen out and the current is entirely due to electrons in the ultra-doped layer. As shown in Fig. 3b,c the relative activation efficiency of P is close to 100% for the sample doped with the low concentration (1 × 10^20 ^cm^−3^) and the carrier mobility is as large as 340 cm^2^/(V·s). The presented mean carrier concentrations for particular doping level were estimated using the measured 2D Hall concentration and the thickness of the doped layer estimated from SIMS measurements (125 nm for a P concentration of 1 × 10^20 ^cm^−3^, 145 nm for 2 × 10^20 ^cm^−3^, 155 nm for 4 × 10^20 ^cm^−3^ and 6 × 10^20 ^cm^−3^). The carrier concentration measured at 2 K was found to be (1.20 ± 0.06) × 10^20 ^cm^−3^ with a carrier mobility of around 260 cm^2^/(V·s) at a concentration of 2 × 10^20 ^cm^−3^ (activation efficiency about 60%), and (2.20 ± 0.11) × 10^20 ^cm^−3^ at a concentration of 4 × 10^20 ^cm^−3^ with a carrier mobility of around 130 cm^2^/(V·s), respectively (see Fig. 3b). For the highest P concentration (6 × 10^20 ^cm^−3^) the efficiency of the electrical activation of P and the carrier mobility are the lowest of the tested samples, namely 25% and 70 cm^2^/(V·s), respectively. According to the Ge-P phase diagram reported by Olesinski *et al*. the maximum solid solubility limit of P in Ge is limited to 5 × 10^19 ^cm^−3^ at 700 °C^30^. In our case, using millisecond range r-FLA at the same annealing temperature, the obtained electrically active P concentration is much higher than the reported solid solubility limit of P in Ge and is one of the highest ever published for P doped Ge layers. For P concentrations in Ge higher than 2 × 10^20 ^cm^−3^ a large fraction of P atoms cannot be electrically active since P will substitute Ge at the neighboring position forming P-P dimers^19^. The probability of P-P dimer formation in implanted Ge is significantly enhanced by conventional high-temperature annealing due to the strong P diffusion and increases with P concentration. Moreover, all lattice damage and remaining vacancies as well as other defects which act as recombination centres lead to the electron mobility degradation and donor compensation which mainly limits the maximum carrier concentration in doped Ge layers using ion implantation. The high activation efficiency obtained here suggests that after r-FLA the implanted layer is practically defect-free. Sheet resistance values below 60 Ω/sqr and carrier densities higher than 2 × 10^20 ^cm^−3^ ensure low access resistance for nano-electronic devices required for source/drain contacts and make Ge attractive for optoelectronic applications.

### Optical properties of ultra-doped Ge

**Figure 4:**
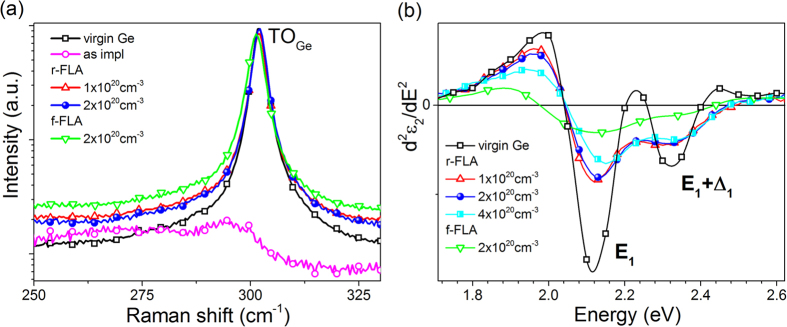
Micro-Raman spectra obtained from P implanted Ge wafers with different P concentrations (**a**) and the high-energy part of the second derivative of imaginary part of the dielectric function (*d*^*2*^*ε*_*2*_(*E*)/*dE*^*2*^) (**b**) after f-FLA and r-FLA.

Figure 4a shows micro-Raman spectra obtained from virgin and P doped Ge at the as-implanted stage and after FLA treatment from either front or rear side. The as-implanted sample exhibits a weak broad phonon mode located at 290 cm^−1^ due to the amorphous Ge layer created during P implantation. The full width at half maximum (FWHM) of the crystalline TO/LO phonon mode (at about 301 cm^−1^) varies from 3.14 cm^−1^ (for virgin Ge) to 3.80 cm^−1^ for P doped Ge after f-FLA. Additionally, the TO peak position of the sample after f-FLA is shifted by 0.60 cm^−1^ towards lower wavenumber. The red shift and the significant broadening of the TO phonon mode indicate the existence of a strongly disordered or polycrystalline Ge layer. This is in agreement with the presented RBS channeling data (shown in Fig. 2a). The same phenomenon was observed by Heera *et al*. in Ge wafers implanted with Ga followed by f-FLA^24^.

Spectroscopic ellipsometry represents a simple method to investigate the band structure of semiconductors. However, due to the low crystalline quality of semiconductors modified by ion implantation, the signatures of the main critical points are often smeared out^31^. Figure 4b shows the second derivative of the imaginary part of the dielectric function taken at an incident angle of 80 degree from a virgin Ge wafer and from Ge samples doped with different P concentrations after r-FLA and f-FLA. The changes in the ellipsometric spectra are caused by the modification of the interband transitions in the vicinity of critical points in the Brillouin zone. The second derivatives of the high-energy part of the dielectric function show two peaks at about 2.12 and 2.30 eV corresponding to E_1_ and E_1_ + Δ_1_ transitions near the Λ point, respectively. In contrast to ion implanted Ge followed by other annealing methods such as nanosecond laser annealing^31^ and f-FLA, in the r-FLA treated samples all critical points are clearly resolved confirming the high-quality of the ultra-doped *n*-type Ge layer. Moreover, the ellipsometric data clearly show a blue shift of the critical points with increasing P concentration. The E_1_ critical point shifts by 35 meV upon increasing the doping of Ge to (2.20 ± 0.11) × 10^20 ^cm^−3^. The blue-shift of the critical points with increasing P doping above 2 × 10^20 ^cm^−3^ is due to the compressive strain caused by the incorporation of P into the lattice of the Ge crystal. During epitaxial regrowth of the implanted layer under r-FLA, the P atoms having a smaller radius than Ge cause an uniaxial lattice compression of the doped layer along [001]. The same effect was observed in Si implanted with B^32^. The critical points observed in the ellipsometry spectrum obtained from the f-FLA sample smear out which is typical for a low crystalline quality or polycrystalline semiconductors.

**Figure 5:**
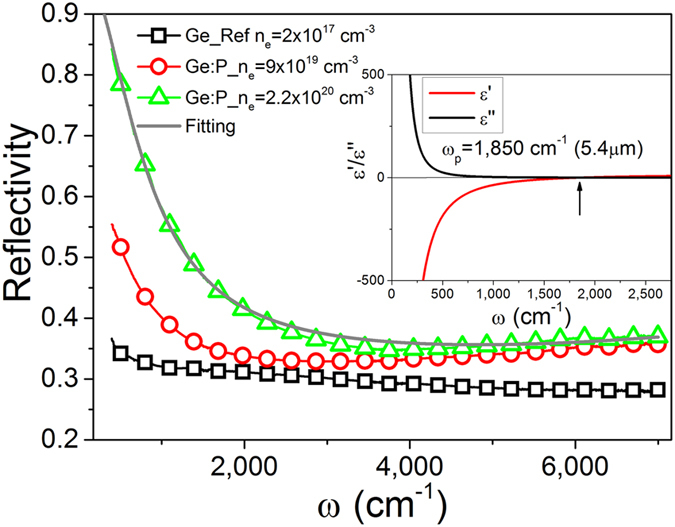
Room-temperature reflectivity measurements from a lightly doped *n*-type Ge (2 × 10^17 ^cm^−3^) wafer and ultra-doped *n*-type Ge thin films after r-FLA 61.5 Jcm^−1^ with active carrier concentrations at 2 K of around 9 × 10^19 ^cm^−3^ and 2.2 × 10^20 ^cm^−3^, respectively. The layer thickness of ultra-doped Ge is around 140 nm and the curves are shown with 0.02 offset for clarity. The grey fitting curve was obtained using the Drude model. The inset shows the real and imaginary part of the dielectric function calculated for the sample with n of 2.2 × 10^20 ^cm^−3^ (*ε′*(ω_p_) = 0 for ω_p_ = 1,850 cm^−1^). The plasma frequency for n = 9 × 10^19 ^cm^−3^ is ω_p_ = 1,175 cm^−1^.

The mid-infrared absorption in ultra-doped Ge was investigated using Fourier transform infrared (FTIR) spectroscopy in the near and mid-infrared spectral range (from 400 to 7000 cm^−1^). Figure 5 shows the reflectivity spectra taken from commercial *n*-type Ge wafer (open squares – Ge-Ref) and ultra-doped Ge layers after r-FLA treatment (open circle – n_e_ = 9 × 10^19 ^cm^−3^ and open triangular n_e_ = (2.20 ± 0.11) × 10^20 ^cm^−3^). The effective carrier concentration in the implanted samples measured by Hall Effect was found to be around (9.00 ± 0.05) × 10^19 ^cm^−3^ and (2.20 ± 0.11) × 10^20 ^cm^−3^ at 2 K and (1.30 ± 0.06) × 10^20 ^cm^−3^ and (3.20 ± 0.16) × 10^20 ^cm^−3^ at 300 K. Prior to the measurements, the reference spectrum was recorded on a gold mirror in vacuum at room-temperature with the same optical alignment which was used to detect the reflectivity from Ge. Spectra presented in Fig. 5 are obtained by dividing the sample spectrum by the reference spectrum. Although the doped layer was only about 140 nm the shift of the plasma edge to higher frequencies with increasing doping concentration is clearly visible. According to theory, the plasma frequency edge is proportional to n^0.5^ and (m^*^)^−0.5^, where n is the carrier concentration and m^*^ is the electron effective mass, and should exhibit a blue shift with increasing n^9^. The plasma frequency ω_p_ for a given doping level and particular material can be represented according to:


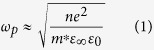


where ε_∞_ and ε_0_ are the high-frequency dielectric constant (for ultra-doped *n*-type Ge ε≈16) and the permittivity of free space, respectively. e is the elementary charge. The plasma frequency ω_p_ taken from the real part of the dielectric function (*ε′* = 0) is 1,850 cm^−1^ (ω_p_ = 5,400 nm) (see the inset in Fig. 5) which is the highest value ever published for *n*-type Ge. To calculate the real and imaginary part of dielectric function we have used the Drude model where: ε_∞_ = 15.04, damping factor 63.35 cm^−1^ and σ_DC_: 13,400 (Ωcm)^−1^.

## Discussion

**Figure 6:**
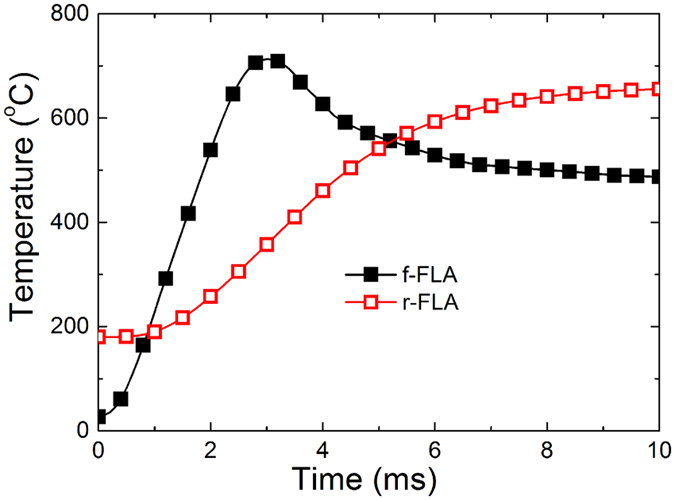
Simulated temperature distribution at the implanted surface during 3 ms FLA with an energy density of 62 Jcm^−2^ from the front side (black curve) and from the rear side (red curve). In the case of rear-side annealing, prior to the flash, the sample was preheated to 180 °C for 30 s.

The absorbed energy delivered to the sample during the FLA process depends on the overall optical absorption and reflection of the investigated system. Averaged over the flash-lamp spectrum, the absorption of amorphous Ge is about 24% higher than that of crystalline Ge. Moreover, the limited ability of a material to dissipate heat by heat conduction will lead to a corresponding strong temperature gradient over the wafer thickness. Therefore, in order to achieve the same temperature within the implanted layer, samples flashed from rear-side were preheated up to 180 °C prior to the flash. Finally, the temperature within the implanted region was about 700 °C for both cases (see Fig. 6). Although the maximum temperature within the implanted layer is roughly the same for both types of annealing, the temperature as a function of time within the implanted layer varies significantly. After f-FLA the implanted layer is kept at a temperature above 600 °C for about 2 ms while after r-FLA the implanted layer is at a temperature higher than 600 °C for a much longer time (see Fig. 6). This has a strong influence on the distribution of dopants in Ge, especially on the fast diffuser phosphorus and on the recrystallization process during the sub-second flash annealing. In order to explain the recrystallization mechanism of an implanted Ge layer during f-FLA, the different values of the activation energy and the pre-factor for seed nucleation and solid phase epitaxy as well as the temperature gradient within the amorphous layer and the speed of heat dissipation have to be taken into account^30^,^31^,^32^,^33^,^34^,^35^,^36^,^37^,^38^. Moreover, the incubation time of crystalline seeds at the surface for the random nucleation has to be considered. According to the reported data from calorimetric studies, e.g. in refs 33,37, in our case the incubation time of crystalline seeds and the recrystallization speed are expected to be in the range of 1 μs and 1 × 10^−4 ^m/s, respectively. On the other hand, L. Nikolova *et al*. have shown that during ns-range laser annealing the incubation time for seed nucleation is as short as 20 ns, that the a-Ge/c-Ge interface moves with a speed of 20 m/s, and that the activation energy is 1.4 eV^38^. For the activation energy of solid phase epitaxy (1.09 eV) a similar value was reported by M. Posselt and A. Gabriel using molecular-dynamics simulations^39^. The present f-FLA method is more similar to pulsed laser annealing than to the conventional rapid thermal annealing or furnace annealing. Moreover, it appears that the incubation time for nucleation and activation energy for the same mechanism is strongly temperature dependent. In our case, during f-FLA we considered both the temperature gradient within the implanted layer and a thermally activated incubation time which delays the onset of solid phase epitaxy from the surface. During f-FLA the energy deposited to the sample surface is absorbed within the first 30–50 nm of the implanted Ge layer leading to a temperature gradient within the amorphous layer. If the amorphized Ge layer is thicker than 100 nm, in case of f-FLA the temperature difference between the surface and the a-Ge/c-Ge interface is large enough to favour recrystallization from the surface via seed nucleation. As a consequence, the poly-crystalline Ge layer propagates towards the crystalline substrate together with heat dissipation because the heat dissipation in a-Ge is much faster than the movement of the crystalline/amorphous (c-Ge/a-Ge) interface at 700 °C^40^.

**Figure 7:**
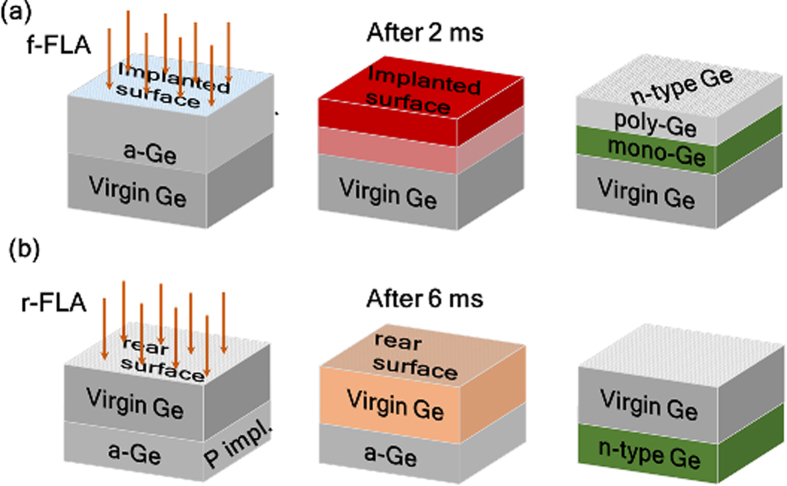
Schematic representation of the recrystallization mechanism of ion implanted Ge during f-FLA (**a**) and r-FLA (**b**).

The deep a-Ge/c-Ge interface gets hot enough for the epitaxial regrowth of the implanted layer towards the surface before the implanted layer is fully recrystallized via seed nucleation (see Fig. 7a). As a result, during flash annealing from the front side two different recrystallization mechanisms take place: recrystallization via seed nucleation and solid phase epitaxy, as schematically shown in Fig. 7a. Interestingly, the snow-plough effect was observed only within the layer regrown via seed nucleation (marked by the arrow in Fig. 2b)^41^,^42^,^43^. Due to this effect the surplus of P is pushed ahead reducing slightly the peak concentration. Within the epitaxially regrown layer no P diffusion was detected (see Fig. 2b). In this particular case, the polycrystalline/single crystalline interface is located at about 115 nm below the surface.

Figure 7b shows schematically the recrystallization process during r-FLA. Because the highest temperature is always obtained at the flashed side, the heat is transferred through the whole wafer to the implanted layer. At such conditions the a-Ge/c-Ge interface gets hot first and the implanted amorphous layer recrystallizes epitaxially. The a-Ge/c-Ge interface has a temperature of about 600 °C after 6 ms during r-FLA. As can be seen in Fig. 6 the temperature within the implanted layer drops below 500 °C within 5 ms after f-FLA and needs more than 20 ms in the case of r- FLA. As a consequence, samples annealed from the rear-side are single crystalline up to the surface and, rather than the snow-plough effect, a slight in-depth diffusion of P was observed (see Fig. 2b). This diffusion leads to the formation of a box-shaped profile of P within the doped layer.

In summary, a very high n-type doping level of 2.2 × 10^20 ^cm^−3^ was achieved in Ge by a combination of ion implantation and r-FLA. In contrast to f-FLA, the implanted Ge layer is single crystalline and practically free of defects after r-FLA. In this case the diffusion of P and the snow plough effect for implanted elements is mainly suppressed. In addition, the plasma frequency can be tuned up to 1,850 cm^−1^ (5,400 nm). The details of the complex crystallization mechanism, especially the balance between the recrystallization speed via solid phase epitaxy and the incubation time followed by nucleation, is still under discussion.

## Methods

### Sample preparation

The (100) oriented undoped Ge wafers with a thickness of 300 μm were implanted with P ions with an energy of 60 keV and to a maximum concentration of 6 × 10^20 ^cm^−3^. The amorphous layer formed during ion implantation is about 200 nm thick. After ion implantation Ge wafers were annealed by front-side FLA (f-FLA) for 3 ms with an energy density up to 61.5 J/cm^2^ which corresponds to a maximum surface temperature of about 700 °C. The same FLA system was used for rear-side annealing. The maximum flash energy was also in the range of 61.5 J/cm^2^ but prior to r-FLA samples were preheated at 180 °C for 30 s. The vertical temperature distribution within the sample during front and rear side annealing was simulated using in-house software. The detailed description of the model used to simulate the temperature distribution in flashed samples can be found in refs 44,45. Figure 7 shows the corresponding surface temperature within the implanted layer as a function of time. P concentration versus depth profiles were measured by secondary ion mass spectrometry (SIMS) employing a Cameca IMS7f micro-analyzer. A beam of 15 keV Cs^+^ ions was rastered over a surface area of 200 × 200 μm^2^ and secondary ions were collected from the central part of the sputtered crater. Crater depths were measured with a Dektak 8 stylus profilometer, and a constant erosion rate was assumed when converting sputtering time to sample depth. Calibration of the P signal was performed using the as-implanted sample as reference.

The crystallization process of the P implanted and annealed samples were studied using Rutherford backscattering-channelling spectrometry (RBS/C). The RBS/C measurements were performed on the samples before and after annealing using the 1.7 MeV He^+^ beam of the Rossendorf van de Graff accelerator.

### Electrical and optical characterization

The conductivity type and carrier concentration of P implanted samples were determined by Hall Effect measurements using a commercial Lakeshore Hall System with the van der Pauw geometry. Samples were measured in the temperature range from 2 to 300 K with a superconducting magnet. Gold electrodes were sputtered onto the four corners of the square-like samples. The sputtering process also removed the native GeO_x_ layer to some extent. Silver glue was used to contact the wires to the gold electrodes. All contacts are confirmed to be ohmic measuring current-voltage curves at different temperatures. The optical properties were investigated by μ-Raman spectroscopy, ellipsometry and Fourier transform infrared spectroscopy in the near and mid-infrared spectral range. The phonon spectra were determined by micro-Raman spectroscopy in backscattering geometry in the range of 100 to 600 cm^−1^ using a 532 nm Nd:YAG laser with a liquid-nitrogen cooled charge coupled device camera. For the ellipsometry measurements a rotating analyser ellipsometer VASE system (J. A.Woollam Co., Inc., USA) operating at an energy range of 1 to 4 eV was used. The FTIR measurements were performed at RT using a Bruker Vertex 80v FT-IR system. The simulation of the dielectric function was performed with a multilayer model based on transfer matrix method.

## Additional Information

**How to cite this article**: Prucnal, S. *et al*. Ultra-doped *n*-type germanium thin films for sensing in the mid-infrared. *Sci. Rep.* **6**, 27643; doi: 10.1038/srep27643 (2016).
